# Single-Step Methylation of Chitosan Using Dimethyl Carbonate as a Green Methylating Agent

**DOI:** 10.3390/molecules24213986

**Published:** 2019-11-04

**Authors:** Ellen B. Hemming, Anthony F. Masters, Alvise Perosa, Maurizio Selva, Thomas Maschmeyer

**Affiliations:** 1 Laboratory of Advanced Catalysis for Sustainability, School of Chemistry, The University of Sydney, Sydney 2006, Australia; ehem5164@uni.sydney.edu.au (E.B.H.); anthony.masters@sydney.edu.au (A.F.M.); thomas.maschmeyer@sydney.edu.au (T.M.); 2 Department of Molecular Sciences and Nanosystems, Università Ca’ Foscari Venezia, Via Torino 155, 30172 Venezia Mestre, Italy

**Keywords:** trimethyl chitosan, green methylating agents, dimethyl carbonate, *N*-methylation, ionic liquids

## Abstract

*N,N,N*-Trimethyl chitosan (TMC) is one chitosan derivative that, because of its improved solubility, has been studied for industrial and pharmaceutic applications. Conventional methods for the synthesis of TMC involve the use of highly toxic and harmful reagents, such as methyl iodide and dimethyl sulfate (DMS). Although the methylation of dimethylated chitosan to TMC by dimethyl carbonate (DMC, a green and benign methylating agent) was reported recently, it involved a formaldehyde-based procedure. In this paper we report the single-step synthesis of TMC from chitosan using DMC in an ionic liquid. The TMC synthesised was characterised by ^1^H NMR spectroscopy and a functionally meaningful degree of quaternisation of 9% was demonstrated after a 12-h reaction time.

## 1. Introduction

**Figure 1:**
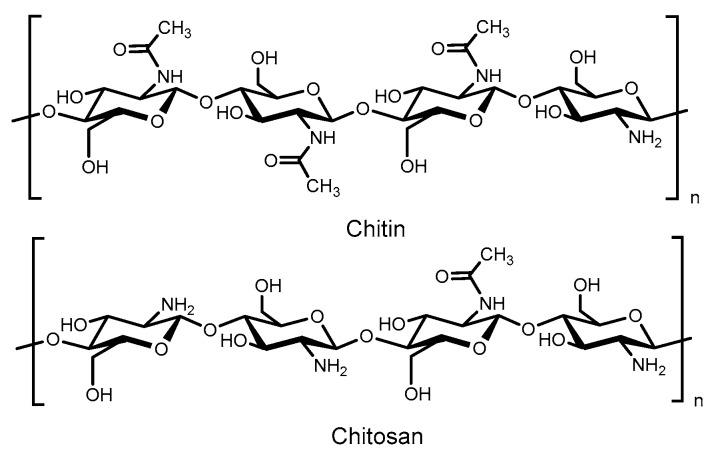
Structures of chitin and chitosan.

Chitin is the second most abundant natural polymer after cellulose and is found as a structural component in the shells and cell walls of a range of organisms, including crustaceans, insects and fungi [1,2,3]. Chitin has a structure similar to that of cellulose, except for a different substituent at the C2 of the pyranose ring, and consists of *β*-1,4 linked *N*-acetylglucosamine and glucosamine units, with the number of glucosamine molecules between 5% and 10% depending on the source (Figure 1) [4]. 

Although potentially advantageous in several fields due to its biocompatibility, biodegradability and nontoxicity, chitin is insoluble in all common solvents including water, which causes a major issue in terms of practical applications [3,5]. A simple solution to overcome this problem is to use chitosan, which is derived from the partial deacetylation of chitin, specifically when the remaining degree of acetylation (i.e., number of *N*-acetylglucosamine units) is less than 50% (Figure 1, bottom) [1,5]. 

Chitosan preserves most of the properties of chitin but, unlike the latter, it is somewhat soluble in acidic aqueous solutions (at least 1 g/L at pH ≤6, for a molecular weight of ca 400 kDa) [1,3,6,7,8]. This improved solubility has fuelled an extended study of chitosan as a feedstock for biomedical, pharmaceutical, food and textile industries serving a variety of applications spanning from drug and gene delivery to heavy metal extraction, tissue engineering and development of antimicrobial and antitumour agents [3,4,7,8]. Due to this huge potential, a highly desirable target to further expand the utilisation of chitosan is its selective functionalisation to synthesise derivatives with improved solubility in water and/or conventional organic solvents [6,7,9,10]. A wide range of methods have proven to be successful for this purpose including the acylation, alkylation, sulfonation and phosphorylation of the hydroxyl and/or amine moieties of chitosan [2,10,11]. This paper will focus on the quaternisation of the amine groups, particularly the methylation of the amine group to produce the corresponding *N,N,N*-trimethyl ammonium salt of chitosan (TMC). The introduction of a permanent positively charged group to chitosan vastly increases the water solubility: one of the best results was achieved with TMC 40-10 (% of *N*-trimethylated and *O*-methylated functions of the chitosan, respectively), which was found to be soluble up to 10 g/L over a pH range of 1–13 [4,12,13,14]. As a consequence, the methylated polymer outperforms the properties of the parent chitosan for biomedical applications, displaying better muco-adhesion, enhanced permeation and antibacterial activity [11,15].

The quaternisation of chitosan to produce TMC has been widely reported in the literature. The vast majority of the methods describe the use of conventional methylating agents, either methyl iodide (MeI) or dimethyl sulfate (DMS), with the addition of a base [9,12,15,16,17,18]. The reaction has been also achieved using a mixture of formaldehyde with either sodium borohydride or formic acid.^14^ All these procedures, however, suffer from major drawbacks:

(i) most require multistep reactions to improve yields of TMC or, in the case of reactions involving formaldehyde, initially produce *N,N*-dimethyl chitosan which, in turn, must be further methylated to the desired TMC, generally through an additional reaction with methyl iodide [19,20];
(ii) stoichiometric processes are involved, thereby cogenerating salts that need to be disposed of;
(iii) most importantly, MeI, DMS and formaldehyde are all toxic, corrosive and potentially carcinogenic reagents [21,22]. Clearly, the adverse impact of these strategies offsets the advantage of using a bio-based and biocompatible polymer such as chitosan.

**Scheme 1:**
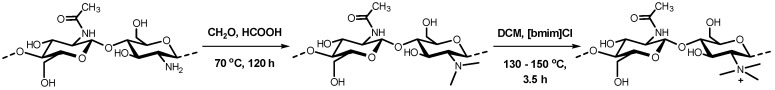
Two-step method reported by Wu et al. for the synthesis of TMC using formaldehyde and DMC as methylating agents [26].

A robust alternative to traditional methylating agents is represented by the nontoxic and biodegradable dimethyl carbonate (DMC) [21,23,24]. Despite its frequent use as a methylating agent, DMC has been described for the methylation of chitosan only in two instances; the first protocol was ineffective for quaternisation [25], the second required a multistep procedure that involved the use of formaldehyde, a toxic reagent [26]. The first method implemented the methylation of chitosan by DMC in the presence of a lipase, as the catalyst, and deep eutectic solvents derived from mixtures of choline chloride and urea or glycerol [25]. However, this procedure led only to the dimethylation of chitosan with no quaternisation observed. Quaternisation was achieved by Wu et al. who combined the use of DMC and an ionic liquid (IL), 1-butyl-3-methylimidazolium chloride ([bmim]Cl), as both the catalyst and the solvent [26]. This method, however, required two steps by which *N,N*-dimethyl chitosan was initially synthesised in a mixture of formaldehyde and formic acid, and subsequently the dimethyl derivative was dissolved in [bmim]Cl and set to react with DMC to afford TMC as the final product (Scheme 1) [26]. 

Although TMC was prepared with a degree of quaternisation of 9%, the involvement of a hazardous reagent, in the form of formaldehyde, posed the same safety and environmental concerns as the protocols discussed above. Although ILs are potentially toxic compounds when ingested, due to their negligible vapour pressure (obviating concerns about environmental solvent loss through evaporation), nonflammability, thermal stability and recyclability, ILs can be safely handled and are acknowledged as green solvents [27,28,29,30,31,32].

As a part of our long-standing interest in the reactivity of DMC for the upgrading of bio-based compounds, the results of Scheme 1 prompted us to inspect more closely the conditions for the methylation of chitosan aimed to implement a fully eco-friendly method [33]. We wish to report herein that the potential of DMC as a quaternisation agent for primary amines [34,35,36] was successfully exploited in the synthesis of TMC. The DMC/[bmim]Cl reaction proceeded smoothly in a one-pot system without the need for additional, toxic methylating agents or the isolation of partially methylated intermediates.

## 2. Results 

**Figure 2:**
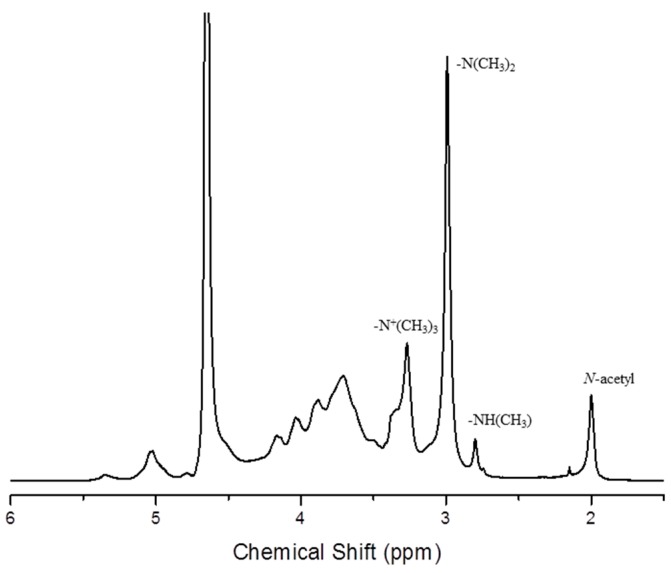
^1^H NMR spectrum of TMC prepared using methyl iodide as the methylating agent recorded in D_2_O at 25 °C.

Chitosan was initially methylated with methyl iodide. The reaction was carried out at 60 °C following an established method that used a mixture of chitosan, KI, MeI and NaOH in a 1:2.6:12.8:0.8 wt ratio, respectively, and N-methyl pyrrolidone (NMP) as a solvent [25]. Since MeI is one of the most powerful methylating agents, this preliminary study aimed to provide a good baseline to assess the effectiveness of DMC as an alternative reagent. The degree of substitution of chitosan was determined by NMR analysis, which is generally accepted as the best technique for this purpose [25,37]. Results shown in Figure 2 illustrate the ^1^H NMR spectrum of the recovered solid after the reaction with methyl iodide. Only the spectral window of interest, between 1 and 6 ppm, is displayed. 

This spectrum is consistent with the formation of a polymer composed of the monomethylated, dimethylated and trimethylated derivatives of amino functions of chitosan, whose corresponding resonances were assigned at δ 2.8, 3.0 and 3.3 ppm, respectively, according to literature data [11,38]. The resonance at δ 2.0 ppm corresponds to the acetyl group of chitosan, which was unaffected by the methylation reaction, and was used as a reference signal [25,38]. A complete assignment of the spectrum can be found in the supplementary data (Table S1).

Thereafter, the reaction of DMC with chitosan was investigated. The choice of the reaction temperature was nontrivial due to the nature of the reactants involved. Chitosan suffers from thermal degradation, which has been reported already at temperatures as low as 60 °C [39,40,41,42]. However, DMC-mediated methylation reactions of most nucleophiles, including phenols, aromatic amines and CH_2_-methylene active compounds, usually take place at temperatures above 120 °C [23,33], although stronger nucleophiles, such as thiols and aliphatic amines, have been occasionally methylated at 90 °C (boiling point of DMC) and, under such milder conditions, specific studies on the methylation of thiols by DMC in ILs have demonstrated that [bmim]Cl was the most efficient solvent [18,20,40,41,43]. 

**Figure 3:**
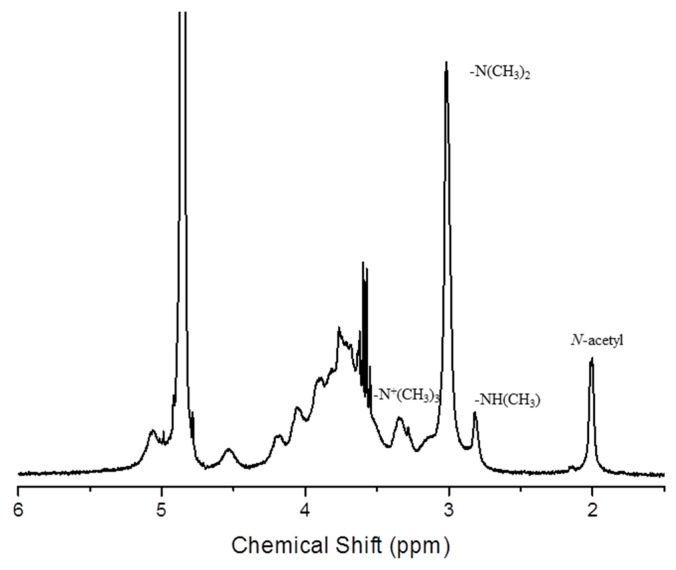
^1^H NMR spectrum of TMC prepared in [bmim]Cl using DMC as the methylating agent recorded in DCl/D_2_O at 25 °C.

These considerations suggested that the refluxing temperature of 90 °C for this system was plausibly suitable for the methylation of the amine groups of chitosan. Moreover, to minimize the evaporation of DMC and the effects of any adventitious oxygen on the thermal degradation of chitosan, a closed reaction vessel (autoclave) under a N_2_ atmosphere was used [44]. Tests were carried out by dissolving chitosan in [bmim]Cl in a 1:10 wt ratio in the presence of a large excess of DMC (5.35:1 wt ratio with respect to the IL and 54:1 with respect to chitosan). The ratio chitosan:IL was determined according to the maximum reported solubility of the biopolymer in [bmim]Cl (10 wt%, see introduction), and was twice the concentration used previously for the alkylation of dimethyl chitosan with DMC [26]. The 54:1 DMC:chitosan ratio was chosen based on the procedure reported by Wu [26] for monomethylation (20:1), increased in the present case considering three consecutive methylation steps. Control experiments were initially conducted for 4 h with the results being reported in Figure 3. 

Thus, the methylation of chitosan by DMC proved feasible, yielding a mixture of the monomethylated, dimethylated and trimethylated amino functions of chitosan, which was qualitatively similar to that of the reaction employing methyl iodide (Figure 2). Additionally, there were no resonances observed between δ 3.4 and 3.5 ppm, which have been widely reported to correspond to the *O*-methylation of chitosan [20,39,45]. This is significant as it suggests that this method is selective for the methylation of the amino functional group. While the quaternisation of the amino functional group increases the solubility of chitosan, *O*-methylation decreases solubility. To compare the performance of the two methylating agents, the degrees of substitutions achieved by MeI and DMC were calculated from the corresponding NMR spectra according to the procedure detailed in the ESI section. Results are reported in Table 1.

As expected, methyl iodide was far more reactive than DMC; in particular, the degree of quaternisation (DQ) to produce fully methylated chitosan (TMC) was estimated to be 20.6%, around three times higher than the corresponding result with DMC (6.7%). This difference in DQs had a significant impact on the solubility of the product: it was observed that the methyl-iodide-derived sample dissolved in neutral aqueous solutions, while the DMC-derived sample required acidic conditions.

**Figure 4:**
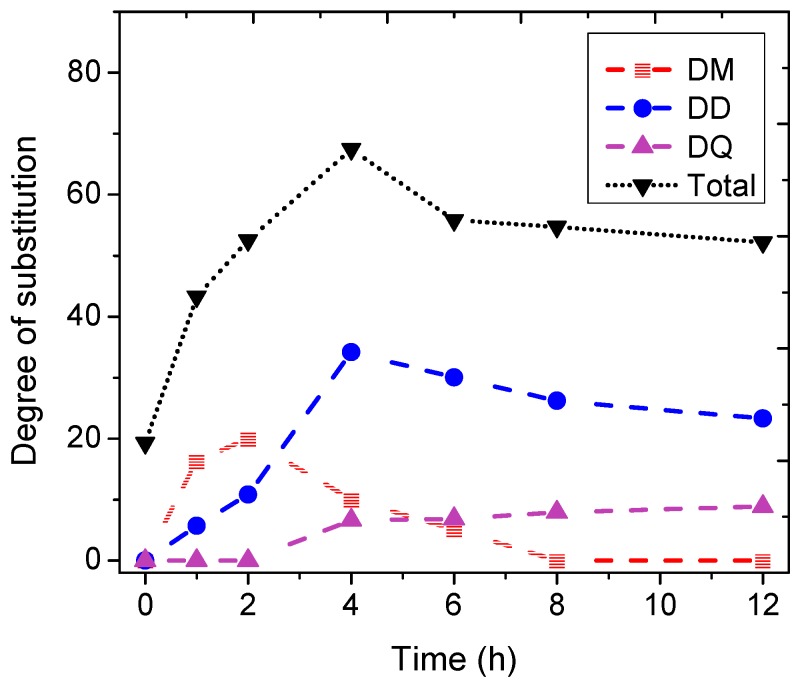
Calculated degrees of monomethylation (DM, 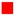), dimethylation (DD, 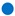) and quaternisation (DQ, 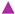), and total degree of substitution (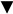) for TMC prepared using DMC as the methylating agent with varying reaction times (0–12 h).

To capture a more complete view, the effect of the reaction time on the reactivity of DMC was explored through additional experiments across periods from 1 to 12 h, using otherwise the same conditions. Detailed NMR profiles are shown in Figure S1 of the ESI section, from which the degree of mono-, di- and trimethylation of chitosan was calculated at each time point. Results are plotted in Figure 4. 

Figure 4 clearly highlights the onset and evolution of the subsequent alkylation steps. The reaction initially proceeded with formation of monomethylated chitosan with its concentration peaking after 2 h (red profile). Then, the further methylation of the monosubstituted derivative induced a substantial increase of dimethylated chitosan with a DD up to 34.2% after 4 h (blue profile). A concurrent trimethylation step was finally observed yielding the desired quaternised product (TMC) with an abundance that progressively increased from 6.7% to 8.9% over the time range between 4 and 12 h. This result is numerically identical in terms of the DQ of 9.1% that was achieved by Wu et al. using a two-step method in which the DMC/[bmim]Cl system was coupled to the use of formaldehyde for the synthesis of TMC [26]. It is important to note that Wu et al. completed the methylation reaction at 130–150 °C under atmospheric pressure whereas the reactions presented here were completed under autogeneous pressure, which may account for the similarity in conversions even at the lower reaction temperature of 90 °C [26]. However, across the time series, it was also observed that the total degree of substitution reached a maximum of 67.6% after 4 h and then decreased to 52.2% (black profile). This behaviour was plausibly due to the decomposition of the functionalised chitosan, which was supported by the change observed within the H3–H6 resonance region (δ 3.5–4.3 ppm) in the NMR spectra of reaction products (ESI, Figure S1). The thermal degradation might be induced either by the longer reaction times and/or the presence of [bmim]Cl. Indeed, the latter has been reported as a catalyst for the conversion of chitosan to 5-hydroxymethylfuran at high temperatures (≥150 °C) and has been shown to be capable of degrading other biopolymers, such as cellulose and starch, at temperatures as low as 100 °C [46,47,48]. One proposed mechanism for the thermal degradation of chitosan suggests that the decomposition occurs due to chain scission and crosslinking of the subunits through the amine groups [42]. Furthermore, this degradation is known to be exacerbated by the substitution of the amino groups [49]. This provides a plausible explanation for the decrease observed in the total degree of substitution over 12 h with the methylated fragments more susceptible to degradation. It is worth noting that the reference protocol method by Wu et al. did not discuss any decomposition issues despite the methylation reactions with DMC being carried out at 130–150 °C for 3.5 h [26]. The relevance of this aspect will be the object of future studies.

For the present study, results of Figure 4 suggested that a reaction time of 4 h was a good compromise between the degree of substitution achieved by the process and the degradation of the sample. Under the conditions investigated here, the concept was proved: the trimethylation of chitosan was obtained through a straightforward one-pot alkylation mediated by DMC in the presence of [bmim]Cl as the solvent. Compared to other procedures, the reaction not only proceeded to an extent of ca 7%, only slightly below the previously reported best value (9%), but it avoided the use of additional (toxic) alkylation reagents and the isolation of intermediates, and took place at a considerably lower temperature of 90 °C instead of 130–150 °C. Additionally, the process was chemoselective for the alkylation of N-sites of chitosan, yielding no *O*-methylation at the hydroxyl functions, a circumstance that should lead to more precise tailoring options for the solubilities of the final material [20]. 

To further improve the DQ, additional experiments were carried out by replacing [bmim]Cl with a deep eutectic solvent ChCl:urea as such or in combination with organic/inorganic bases, such as caesium carbonate and DBU (details are in the ESI section). Any such attempt, however, proved unsuccessful. This result is consistent with the statement that [bmim]Cl plays an integral role in dissolving chitosan and making its amine groups available by freeing them from H-bonds, while in parallel stabilising the reaction intermediates through the imidazolium cation [43].

## 3. Materials and Methods

All chemicals were used as received unless stated otherwise: dimethyl carbonate (DMC), chitosan (low molecular weight, Mw 50–190 kDa, degree deacetylation ≥75%), potassium iodide, sodium hydroxide, 1-chlorobutane, acetonitrile, ethyl acetate, ethanol, deuterium chloride (35 wt% in D_2_O), deuterium oxide (all Sigma-Aldrich), methyl iodide (Janssen), 1-methyl-2-pyrrolidinone (NMP, Fluka) and toluene (Carlo Erba). 1-Methylimidazole was purified before use by vacuum distillation. 

NMR spectra were recorded at 298 K on a Bruker AVANCE 300 spectrometer operating at 300.15 MHz. The samples were dissolved in either D_2_O or DCl/D_2_O and peaks were referenced to the *N*-acetyl peak at 2.0 ppm.

### 3.1. Methylation of Chitosan Using Dimethyl Carbonate

A 25 mL stainless-steel autoclave was charged with chitosan (0.1 g), [bmim]Cl (1.0 g) and dimethyl carbonate (5.0 mL) and flushed with nitrogen by briefly evacuating and then backfilling with nitrogen three times. The mixture was stirred at 90 °C for the desired length of time. The mixture was cooled to room temperature and ethanol (~10 mL) was added. The mixture was filtered and the solid was washed with hot ethanol to produce the methylated chitosan (~0.099 g).

### 3.2. Methylation of Chitosan Using Methyl Iodide

The following procedure was adapted from Hamman and Kotzé [50]. A mixture of chitosan (0.5 g) and potassium iodide (1.3 g) in NMP (20.0 mL) was stirred at 60 °C for 1.5 h. An aqueous sodium hydroxide solution (2.8 mL, 15% *w*/*v*) and then methyl iodide (2.8 mL) were added and the solution was stirred at 60 °C for 2 h. The product was precipitated by the addition of ethanol and isolated by centrifugation. After washing with ethanol, the product was dissolved in an aqueous sodium chloride solution (40 mL, 5% *w*/*v*). The product was precipitated by the addition of ethanol, centrifuging to isolate and dried to produce the methylated chitosan (0.498 g).

Fresh [bmim]Cl was synthesised by the reaction of methylimidazole and butyl chloride as detailed in the ESI section.

## 4. Conclusions

The procedure reported demonstrates that experimental conditions for the methylation of chitosan with dimethyl carbonate as the alkylation agent and [bmim]Cl as the solvent can be successfully tuned, in particular by changing the temperature, time and concentration, to implement an unprecedented one-pot protocol. Synthetic limitations still exist in comparison to classical methylation reagents, such as methyl iodide, by which the degree of quaternisation (DQ = 20.6%) of chitosan is almost tripled with respect to DMC (6.7–8.9%). However, this study offers a significant improvement towards the implementation of a fully green method for the upgrading of chitosan, involving only catalytic reactions with no metals or side products to be disposed of (except for methanol and CO_2_, which have the potential to be converted back to DMC), and no toxic reagents [22]. These advantages become even more relevant in view of using a functionalised bio-based material, such as methylated chitosan, for biomedical applications where residual traces of hazardous contaminants may be fatal.

Based on these promising results, future efforts will be focused on preventing or limiting the thermal degradation of chitosan observed over prolonged reaction times.

## Figures and Tables

**Table 1:** Calculated degrees of monomethylation (DM), dimethylation (DD), quaternisation (DQ) and acetylation (DAc) for TMC prepared using different methylating agents.

Methylating Agent	DM (%)	DD (%)	DQ (%)	DAc (%)	Total (%) *
**Methyl Iodide**	6.9	47.3	20.6	15.5	90.3
**DMC**	10.0	34.2	6.7	16.7	67.6

* Sum of DM, DD, DQ and DAc.

## Supplementary Materials

The following are available online at https://www.mdpi.com/1420-3049/24/21/3986/s1, Table S1: Chemical shifts of the protons of TMC, Figure S1: ^1^H-NMR spectra of TMC prepared in [bmim]Cl using DMC as the methylating agent with varying reaction times (0–12 h), Figure S2: 1H-NMR spectra of TMC prepared in ChCl:urea DES using DMC as the methylating agent and either cesium carbonate or DBU as the base.
