# Supplementary material for: Single-Step Methylation of Chitosan Using Dimethyl Carbonate as a Green Methylating Agent
Source: Molecules. 2019 Nov 4;24(21):3986. doi: 10.3390/molecules24213986 (PMC6864761; doi:10.3390/molecules24213986)
Supplement: Supplementary file 1 [file molecules-24-03986-s001.pdf]

# Supporting Information

for

# Single step methylation of chitosan using dimethyl carbonate as a green methylating agent

E. B. Hemming,[a,b] A. F. Masters,[a] A. Perosa,[b,*] M. Selva,[b,*] and T. Maschmeyer[a]

[a] Laboratory of Advanced Catalysis for Sustainability, School of Chemistry, The University of Sydney, Sydney 2006, Australia.
[b] Department of Molecular Sciences and Nanosystems, Università Ca' Foscari Venezia, Via Torino 155, 30172 Venezia Mestre (Italy). E-mail: alvise@unive.it, selva@unive.it

## Experimental

*Materials*

All chemicals were used as received unless stated otherwise. 1-Chlorobutane, acetonitrile, ethyl acetate, urea, caesium carbonate (all Sigma-Aldrich), 1,8-diazabicyclo[5.4.0]undec-7-ene (DBU, Alfa-Aesar) and toluene (Carlo Erba). 1-Methylimidazole was purified before use by vacuum distillation. Choline chloride (Sigma-Aldrich) was dried under vacuum at 70 °C before use.

*Synthesis of 1-butyl-3-methylimidazolium chloride ([bmim]Cl)*

The following procedure was adapted from Riisager and Bosmann.[1] A solution of 1-methylimidazole (10.0 mL, 0.13 mol) and 1-chlorobutane (19.5 mL, 0.19 mol) in toluene (13 mL) was stirred under nitrogen at 110 °C for 24 h. The mixture was cooled and allowed to separate into two phases. The toluene phase was decanted and the bottom ionic liquid phase was washed with toluene (3 × 15 mL) before drying with a rotary evaporator to give the crude [bmim]Cl as a clear viscous liquid. The product was

recrystallised from acetonitrile and ethyl acetate and dried at 40 °C to produce white crystals (17.9 g, 82%). **$^1$H NMR** (400 MHz, $CDCl_3$): δ 10.53 (1H, s, NC**H**N), 7.61 (1H, m, $CH_3$NCHC**H**N), 7.45 (1H, m, $CH_3$NC**H**CHN), 4.25 (2H, t, $^3J_{HH}$ = 7.2 Hz, NC**$H_2$**$CH_2CH_2CH_3$), 4.04 (3H, s, NC**$H_3$**), 1.82 (2H, m, $NCH_2$C**$H_2$**$CH_2CH_3$), 1.29 (2H, m, $NCH_2CH_2$**$CH_2$**$CH_3$), 0.87 (3H, t, $^3J_{HH}$ = 8.0 Hz, N $CH_2CH_2CH_2$C**$H_3$**) ppm. **$^{13}$C[$^1$H] NMR** (75 MHz, $CDCl_3$): δ 137.9, 123.7, 122.0, 49.7, 36.5, 32.1, 19.4, 13.4 ppm.

*Analysis of 1H MNR spectra recorded after the methylation of chitosan with both MeI and DMC (conditions of Figures 2 and 3 and Table 1)*

**Table S1**. Chemical shifts of the protons of TMC.[2]

| Proton environment | Chemical Shift (ppm) |
|---|---|
| **Acetyl protons** | 2.0 |
| **Protons of –$NCH_3$** | 2.8 |
| **Protons of –$N(CH_3)_2$** | 3.0 |
| **H2** | 3.1 |
| **Protons of –$N^+(CH_3)_3$** | 3.3 |
| **H3, H4, H5, H6, H6′** | 3.5–4.3 |
| **H1** | 4.9–5.4 |

*Estimation of degree of chitosan substitution by $^{1}H$ NMR*

The degrees of substitution for the samples were estimated from the $^{1}H$ NMR spectra using the following formulae:

$$Degree\ of\ acetylation\ (DA) = \left[\frac{\frac{1}{3} \times I_{acetyl}}{\frac{1}{5} \times I_{3-6}}\right] \times 100$$

$$Degree\ of\ quaternisation\ (DQ) = \left[\frac{\frac{1}{9} \times I_{N(CH3)3}}{\frac{1}{5} \times I_{3-6}}\right] \times 100$$

$$Degree\ of\ dimethylation\ (DD) = \left[\frac{\frac{1}{6} \times I_{N(CH3)2}}{\frac{1}{5} \times I_{3-6}}\right] \times 100$$

$$Degree\ of\ monomethylation\ (DM) = \left[\frac{\frac{1}{3} \times I_{NH(CH3)}}{\frac{1}{5} \times I_{3-6}}\right] \times 100$$

Where $I_{3\text{-}6}$ is the sum of integrals for the five protons H3, H4, H5 and H6. $I_{acetyl}$, $I_{N(CH3)3}$, $I_{N(CH3)2}$ and $I_{NH(CH3)}$ refer to the integral of the acetyl, trimethyl, dimethyl or monomethyl groups, respectively.

*The effect of the reaction time on the reactivity of DMC*

Experiments were carried out under the conditions of Figure 3, except for the reaction time which was set to vary between 1 and 12 hours. Results are reported in Figure S1.

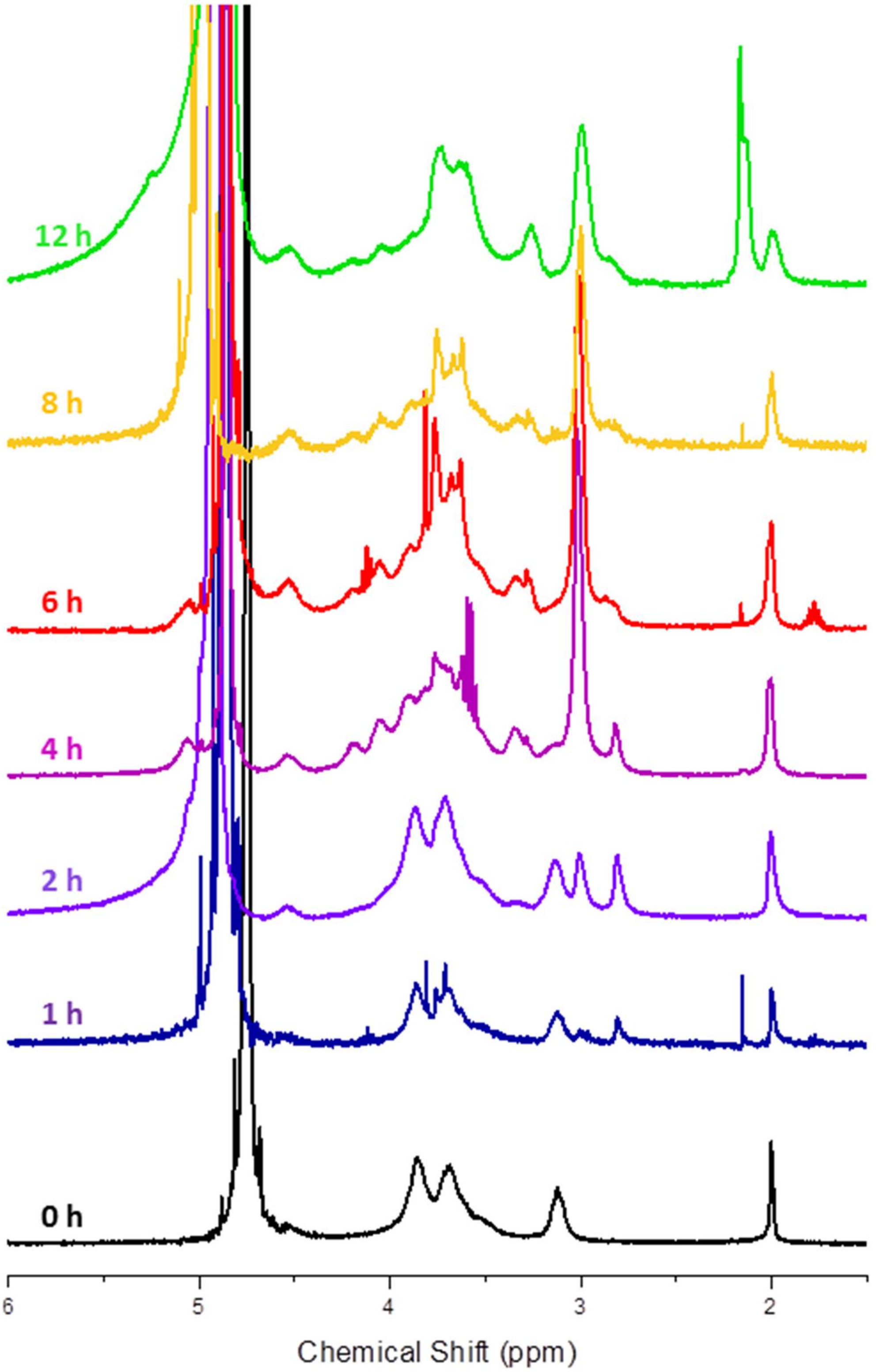


**Figure S1.** $^{1}$H NMR spectra of TMC prepared in [bmim]Cl using DMC as the methylating agent with varying reaction times (0-12 h).

*Methylation of chitosan with DMC in DESs attempts.*

Experiments were conceived based on the results described for the methylation of chitosan with DMC in the presence of a lipase catalyst and deep eutectic solvents (DESs).[2] To this purpose a (ChCl:urea) was prepared.

*Synthesis of choline chloride:urea (ChCl:urea) DES*

The following synthetic method was adapted from Singh *et al.*[3] A mixture of choline chloride (4.0 g, 28.6 mmol) and urea (3.4 g, 57.3 mmol) was stirred at 80 °C under an atmosphere of nitrogen for 1 h to produce a clear viscous liquid (7.4 g, 99%). **$^{1}$H NMR** (400 MHz, MeOD-$d_4$): δ 4.76 (8H, s, -$NH_2$), 3.94 (2H, m, C**$H_2$**OH), 3.44 (2H, m, C**$H_2$**), 3.16 (9H, s, -$N^+$(C**$H_3$**)$_3$) ppm. **$^{13}$C[$^{1}$H] NMR** (75 MHz, MeOD-$d_4$): δ 162.1, 68.2, 56.3, 53.9 ppm.

*Reaction of chitosan using (ChCl:urea) DES as the solvent*

A 25 mL stainless-steel autoclave was charged with chitosan (0.1 g), ChCl:urea (1.0 g), dimethyl carbonate (5.0 mL) and either caesium carbonate (0.1 g) or DBU (0.1 g). The autoclave was flushed with nitrogen by briefly evacuating and then backfilling with nitrogen three times. The mixture was stirred at 90 °C for 4 h, before cooling to room temperature and adding ethanol (~10 mL). The mixture was filtered and the solid was washed with hot ethanol.

Any of such attempts proved unsuccessful with no methylated products observed. Results are shown in Figure S2, which reports NMR spectra recorded after 4 hours of reaction.

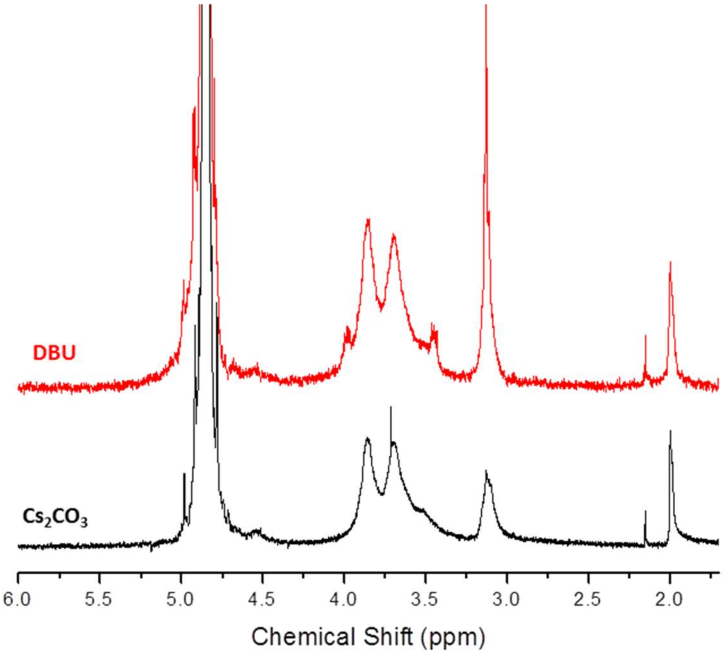


**Figure S2.** $^1$H NMR spectra of TMC prepared in ChCl:urea DES using DMC as the methylating agent and either cesium carbonate or DBU as the base.

## References

(1) Riisager, A.; Bosmann, A.: Ionic Liquids as Benign Solvents for Sustainable Chemistry. In *Experiments in Green and Sustainable Chemistry*; Roesky, H. W., Kennepohl, D., Eds.; Wiley-VOH: Weinheim, 2009; pp 108-113.

(2) Bangde, P. S.; Jain, R.; Dandekar, P.: Alternative Approach to Synthesize Methylated Chitosan Using Deep Eutectic Solvents, Biocatalyst and "Green" Methylating Agents. *ACS Sustainable Chem. Eng.* **2016**, *4*, 3552-3557.

(3) Singh, B.; Lobo, H.; Shankarling, G.: Selective N-Alkylation of Aromatic Primary Amines Catalyzed by Bio-catalyst or Deep Eutectic Solvent. *Catal. Lett.* **2011**, *141*, 178-182.
